# Bioavailability-Enhanced Curcumin (CurcuBoost®) as Adjunct Therapy Improves Symptoms in Knee Osteoarthritis: A Randomized Clinical Trial

**DOI:** 10.7759/cureus.112993

**Published:** 2026-07-20

**Authors:** Lokesh Bansal, Devesh Kumar

**Affiliations:** 1 Department of Orthopedics, Panchsheel Hospital Pvt. Ltd., Delhi, IND; 2 Department of Clinical Operations, IR Innovate Research Pvt. Ltd., Noida, IND

**Keywords:** adjunct therapy, bioavailability-enhanced curcumin, curcuboost®, functional outcomes, osteoarthritis, quality of life, womac

## Abstract

Objectives

This study aimed to evaluate the efficacy, safety, and tolerability of the bioavailability-enhanced curcumin formulation CurcuBoost® as an adjunct to standard of care (SOC) in subjects with knee osteoarthritis (OA).

Methods

In this randomized, open-label, parallel-group trial, adults aged 40-75 years with radiographically confirmed knee OA (Kellgren-Lawrence grade II-III) were randomized (1:1) to receive CurcuBoost® 500 mg once daily + SOC or SOC alone for eight weeks. Analyses were conducted in the modified intention-to-treat (mITT) population.

Results

All 50 participants completed the study. At week 8, CurcuBoost® + SOC resulted in significantly greater reductions in Western Ontario and McMaster Universities Osteoarthritis Index (WOMAC) pain compared with SOC alone (mean ± SD: 4.68 ± 1.65 vs 7.40 ± 2.18; p < 0.0001), corresponding to a 65.2% versus 44.0% reduction from baseline. Significant improvements favoring CurcuBoost® were also observed in WOMAC physical function (-59.1% vs -38.9%; p < 0.0001), WOMAC total score (-59.9% vs -40.7%; p < 0.0001), and Visual Analog Scale (VAS) pain score (-61.5% vs -39.5%; p < 0.0001). Outcome Measures in Rheumatology-Osteoarthritis Research Society International (OMERACT-OARSI) responder rates at week 8 were higher with CurcuBoost® (80% (n = 20) vs 48% (n = 12); p = 0.0184). Improvements in the EuroQol 5-Dimension questionnaire (EQ-5D) and global assessments were also significantly greater (all p < 0.01) in the CurcuBoost® + SOC group. No treatment-related serious adverse events (SAEs) were reported.

Conclusion

CurcuBoost® as an adjunct to SOC significantly improved pain, function, and quality of life in knee OA with a favorable safety profile, supporting its role as an effective complementary therapeutic option.

## Introduction

Osteoarthritis (OA) is a highly prevalent chronic joint disorder affecting approximately 528 million people worldwide, predominantly adults aged ≥40 years, and is a leading cause of pain, disability, and reduced quality of life [1]. Knee OA accounts for most symptomatic cases due to the joint’s high mechanical load and is characterized by progressive cartilage degeneration, synovial inflammation, subchondral bone changes, stiffness, pain, and functional impairment [1,2]. Pain and mobility limitations contribute substantially to global disability, particularly in aging populations [2,3]. Current management focuses on symptom relief and functional improvement through non-pharmacological and pharmacological approaches, including physical therapy and nonsteroidal anti-inflammatory drugs (NSAIDs) [4,5]. Although effective, long-term NSAID use is limited by gastrointestinal, renal, and cardiovascular adverse effects, especially in older adults and those with comorbidities, and does not alter disease progression [4-6]. Safe and effective adjunctive therapies are therefore needed [4,5].

Curcumin, the principal bioactive compound of *Curcuma longa*, has gained attention in OA management for its anti-inflammatory, antioxidant, and chondroprotective effects [7-9]. Preclinical studies show that curcumin modulates key pathways in OA pathogenesis, including inhibition of nuclear factor-κB signaling, suppression of pro-inflammatory cytokines (tumor necrosis factor-α, interleukin-1β), downregulation of cyclooxygenase-2, and reduction of oxidative stress and matrix metalloproteinase activity [8-10]. Clinical trials and meta-analyses report significant improvements in pain and function, measured by the Visual Analog Scale (VAS) and the Western Ontario and McMaster Universities Osteoarthritis Index (WOMAC) [10-12]. An umbrella meta-analysis demonstrated reductions in VAS pain and improvements in WOMAC total and subscale scores [10]. Some analyses suggest comparable efficacy to NSAIDs with fewer adverse events (AEs), supporting a favorable safety profile [2,5].

However, conventional curcumin has poor oral bioavailability due to low solubility, rapid metabolism, and limited absorption, resulting in low systemic exposure and variable clinical effects [13,14]. To overcome these limitations, enhanced formulations such as micelles, nanoparticles, phospholipid, and cyclodextrin complexes have been developed to improve solubility, stability, and absorption [14-17]. Certain formulations achieve 16- to 50-fold greater bioavailability than standard extracts, potentially enabling more consistent therapeutic outcomes [16,17].

CurcuBoost® is a proprietary curcumin formulation designed to enhance curcuminoid absorption and systemic exposure. Given safety concerns with prolonged NSAID use and accumulating evidence supporting curcumin’s efficacy and tolerability, evaluating CurcuBoost® as an adjunct to standard-of-care (SOC) therapy is warranted. The primary objective of this study was to assess the efficacy of CurcuBoost® + SOC in subjects with knee OA, measured by change in WOMAC pain score from baseline to week 8. Secondary objectives included changes in WOMAC stiffness, function, and total scores; Outcome Measures in Rheumatology-Osteoarthritis Research Society International (OMERACT-OARSI) responder responder status; patient-reported outcomes, including EuroQol 5-Dimension (EQ-5D) questionnaire and global assessments; VAS pain; and safety over 8 weeks.

## Materials and methods

Study design and ethical considerations

This was a randomized, open-label, single-center, parallel-group clinical trial conducted between March 6, 2025, and July 8, 2025. The study was approved by the Central Independent Ethics Committee, Pune, Maharashtra, India (IEC Reference no. CIEC190225, dated February 18, 2025) and was conducted in compliance with ICH-E6(R2) Good Clinical Practice, ICMR guidelines (2017), and the Helsinki Declaration of 1975, as revised in 2000. The trial was prospectively registered with the Clinical Trials Registry of India prior to participant enrollment (CTRI No: CTRI/2025/02/081342; registered on: 25/02/2025). All subjects provided written informed consent prior to participation and before undergoing any study-specific procedures.

Study population

Men and women aged 40-75 years were eligible for enrollment in the study if they had a diagnosis of knee OA based on American College of Rheumatology criteria and radiographic Kellgren-Lawrence grade II or III. Eligible patients were required to have moderate-to-severe walking pain in the target knee, defined by a VAS pain score > 40 mm at screening. For patients with bilateral osteoarthritis, the target knee (most symptomatic or more advanced disease) was pre-specified for all assessments. Participants were required to have documented NSAID use for at least 30 days prior to enrollment. Exclusion criteria included recent glucocorticoid or intra-articular therapy, use of slow-acting osteoarthritis disease-modifying supplements, or any medical or psychological condition that could interfere with study compliance or data integrity. The inclusion and exclusion criteria are presented in Table 1.

**Table 1:** Inclusion and exclusion criteria ACR: American College of Rheumatology, NSAID: nonsteroidal anti-inflammatory drug, OA: osteoarthritis, VAS: Visual Analog Scale.

Inclusion criteria	Exclusion criteria
Men and women; age 40-75 years	Recent glucocorticoid therapy
Knee osteoarthritis (ACR criteria)	Recent intra-articular therapy
Kellgren-Lawrence grade II-III	Use of OA disease-modifying supplements
VAS > 40 mm (target knee)	Significant medical conditions
Target knee defined (if bilateral OA)	Significant psychological conditions
NSAID use ≥ 30 days	

Randomization and treatment

Eligible participants were randomized in a 1:1 ratio to receive either CurcuBoost® + SOC or SOC alone using a computer-generated randomization list prepared by an independent biostatistician. Treatment allocation was performed at the baseline/randomization visit (day 1) according to the predefined randomization schedule. Because of the nature of the intervention, the study was conducted using an open-label design, and blinding was not applicable. Participants assigned to the investigational arm received one 500-mg CurcuBoost® capsule orally once daily after meals for 56 ± 2 days in addition to SOC, while participants in the control arm received SOC alone. SOC was administered for the same treatment duration in both groups and consisted of meloxicam or paracetamol, prescribed according to the investigator's clinical judgment and routine clinical practice. The same SOC treatment principles were applied to both study arms. Concomitant medications were prospectively documented using participant diaries and reviewed at each study visit to monitor treatment exposure and compliance. Medication adherence was assessed by diary review and capsule count, with ≥80% dosing considered compliant.

Assessments

Study visits occurred at baseline (day 1), week 4 (day 28 ± 2), and week 8 (day 56 ± 2). At each visit, physical examination, vital signs, assessment of concomitant medications, and AE monitoring were performed. Efficacy assessments included WOMAC pain, stiffness, function subscales, and total score [18], Patient Global Assessment (PGA), Investigator Global Assessment (IGA), VAS pain scale [19], and the EQ-5D questionnaire [20]. OMERACT-OARSI responder status was evaluated at weeks 4 and 8 [21]. Medication adherence was assessed by diary review and capsule count, with ≥80% dosing considered compliant.

Endpoints

The primary efficacy endpoint was the change in WOMAC pain score from baseline (week 0) to week 8. Secondary endpoints included change in WOMAC subscales (pain, stiffness, and function), change in VAS pain score, Patient and Investigator Global Assessments, EQ-5D scores, OMERACT-OARSI responder rate, and incidence of AEs and serious AEs (SAEs).

Safety monitoring

All treatment-emergent AEs (TEAEs) and SAEs were recorded, assessed for severity and causal relationship to treatment, and monitored until resolution or stabilization. SAEs were reported to the sponsor and ethics committee within mandated timelines. Subjects who discontinued treatment prematurely underwent repeat safety assessments, including physical examination and vital signs.

Sample size calculation

A total sample size of 50 participants (25 per group) was considered adequate for this exploratory randomized clinical study. During study planning, the sample size was based on detecting a clinically meaningful improvement in overall WOMAC outcomes. However, in accordance with the study protocol, change in the WOMAC pain subscale from baseline to week 8 was prespecified as the primary efficacy endpoint and served as the basis for the primary efficacy analysis.

Statistical analysis

Statistical analyses were conducted using R statistical software (version 4.4.1, R Foundation for Statistical Computing, Vienna, Austria, https://www.R-project.org/). All statistical analyses were prespecified in the study protocol and performed on both the modified intention-to-treat (mITT) and per-protocol (PP) analysis populations. The mITT population included all randomized participants who received at least one dose of the study medication and had baseline efficacy assessments, whereas the PP population comprised participants who completed the study without major protocol deviations. The primary efficacy analyses were conducted using the mITT population, with supportive analyses performed using the PP population. Missing data for efficacy endpoints were planned to be handled using the last observation carried forward (LOCF) method if required. However, as all randomized participants completed the study and had evaluable outcome data at all scheduled visits, no imputation was required, and the LOCF method was not applied.

Continuous variables, including WOMAC total and subscale scores and VAS pain scores, were summarized using descriptive statistics, including the number of observations, mean, standard deviation (SD), median, minimum, and maximum. Categorical variables, such as responder rates and the incidence of AEs, were summarized using frequencies and percentages. In addition, 95% confidence intervals (CIs) for means were calculated and presented where appropriate. Between-group comparisons for continuous variables were performed using Student’s t-test. For change-from-baseline outcomes, analysis of covariance (ANCOVA) models adjusted for the corresponding baseline outcome values were applied. No additional baseline covariates, including body mass index (BMI), were included in the prespecified analyses. Categorical variables were compared between treatment groups using the chi-square test or Fisher’s exact test, as appropriate. Effect size measures, including relative risks or odds ratios, were calculated for categorical outcomes when relevant. All statistical tests were two-sided, and a p-value < 0.05 was considered statistically significant.

The study was powered to evaluate the primary efficacy endpoint (change in WOMAC pain score at week 8). No formal adjustment for multiplicity was prespecified for analyses of the secondary efficacy endpoints. Accordingly, analyses of secondary outcomes were considered supportive and exploratory, and the corresponding p-values are interpreted descriptively.

## Results

Participant disposition

**Figure 1:**
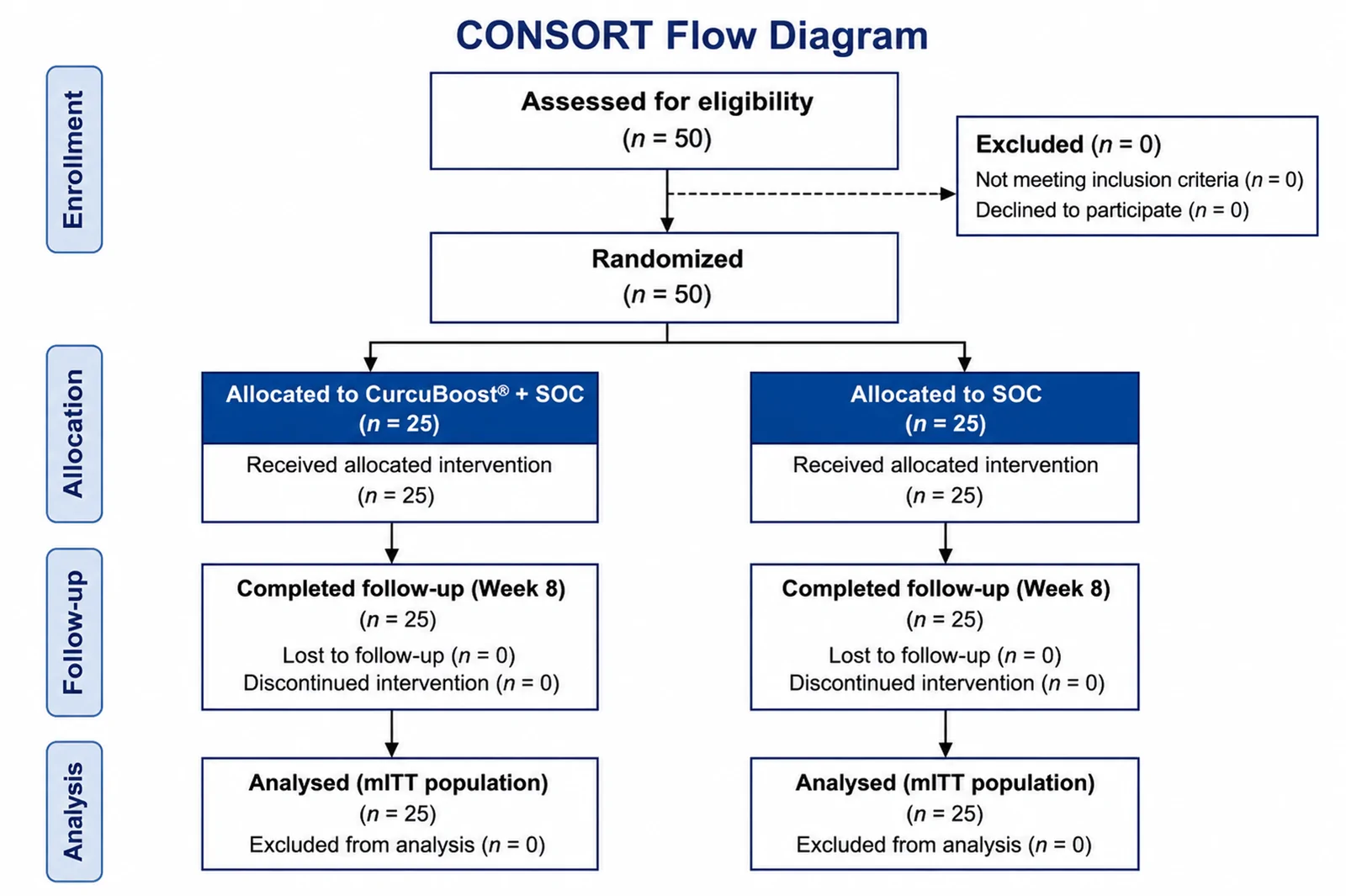
Consort flow diagram of participant screening, randomization, allocation, follow-up, and analysis mITT: modified intention-to-treat, SOC: standard of care.

A total of 50 participants were randomized, with 25 assigned to CurcuBoost® + SOC and 25 to SOC alone. All randomized participants completed the eight-week study, with no withdrawals or losses to follow-up, resulting in a 100% completion rate in both treatment groups. All participants were included in the safety and mITT populations (Figure 1).

Baseline characteristics

Baseline demographic characteristics were generally comparable between groups (Table 2). Mean age, sex distribution, and body weight were similar, whereas baseline BMI was significantly lower in the CurcuBoost® + SOC group than in the SOC group (p = 0.0219).

**Table 2:** Baseline demographic characteristics (mITT population, n = 50) Data presented as mean ± SD and % (n) as specified. p-values < 0.05 were considered statistically significant. BMI: body mass index, mITT: modified intention to treat, SOC: standard of care.

Parameter	CurcuBoost® + SOC (N = 25)	SOC (N = 25)	p-value
Age (years), mean ± SD	55.76 ± 10.12	55.12 ± 8.88	0.8131
Female sex, n (%)	9 (36%)	12 (48%)	0.3900
BMI (kg/m²), mean ± SD	25.78 ± 3.49	28.79 ± 5.28	0.0219
Weight (kg), mean ± SD	68.16 ± 8.87	70.60 ± 10.31	0.3742

Primary efficacy outcome

WOMAC Pain Score

At baseline, WOMAC pain scores were comparable between groups (Table 3).

**Table 3:** WOMAC outcomes (mITT population, n = 50) Data are presented as mean ± SD. p-values < 0.05 for ANCOVA were considered statistically significant. 95% CIs and Cohen's d values were calculated for between-group differences in change scores. "Δ" indicates change from baseline. SOC: standard of care, WOMAC: Western Ontario and McMaster Universities Osteoarthritis Index, mITT: modified intention to treat, ANCOVA: analysis of covariance.

Time point/metric	CurcuBoost® + SOC (n = 25)	SOC alone (n = 25)	Mean difference (95% CI)	Cohen’s d	p-value
WOMAC pain					
Baseline	13.84 ± 2.06	13.52 ± 2.49	-	-	-
Week 4	8.28 ± 2.73	9.84 ± 2.25	-	-	0.0322
Week 8	4.68 ± 1.65	7.40 ± 2.18	-	-	<0.0001
Δ week 4	-5.56	-3.68	-	-	0.0322
Δ week 8	-9.16 ± 2.42	-6.12 ± 2.74	-3.04 (-4.47 to -1.61)	1.17	<0.0001
% Δ week 8	-65.2 ± 13.4	-44.0 ± 18.4	-	-	<0.0001
WOMAC stiffness					
Baseline	7.00 ± 0.91	6.68 ± 0.99	-	-	-
Week 4	4.64 ± 1.33	5.28 ± 1.39	-	-	0.0912
Week 8	3.16 ± 1.14	3.52 ± 1.53	-	-	0.3161
Δ week 4	-2.36	-1.40	-	-	0.0912
Δ week 8	-3.84 ± 1.21	-3.16 ± 1.40	-0.68 (-1.41 to 0.05)	0.52	0.3161
% Δ week 8	-54.8 ± 14.8	-47.4 ± 21.7	-	-	0.1747
WOMAC physical function					
Baseline	60.64 ± 3.99	62.48 ± 3.49	-	-	-
Week 4	40.88 ± 9.33	49.28 ± 10.88	-	-	0.0045
Week 8	24.92 ± 7.71	38.00 ± 11.47	-	-	<0.0001
Δ week 4	-19.76	-13.20	-	-	0.0045
Δ week 8	-35.72 ± 7.07	-24.48 ± 12.20	-11.24 (-16.84 to -5.64)	1.12	<0.0001
% Δ week 8	-59.1 ± 12.1	-38.9 ± 18.9	-	-	<0.0001
WOMAC total score					
Baseline	81.48 ± 5.72	82.68 ± 4.88	-	-	-
Week 4	53.80 ± 11.41	64.40 ± 12.74	-	-	0.0021
Week 8	32.76 ± 8.99	48.92 ± 13.50	-	-	<0.0001
Δ week 4	-27.68	-18.28	-	-	0.0021
Δ week 8	-48.72 ± 8.65	-33.76 ± 13.77	-14.96 (-21.36 to -8.56)	1.30	<0.0001
% Δ week 8	-59.88 ± 10.49	-40.74 ± 16.23	-	-	<0.0001

At week 4, both groups demonstrated reductions in WOMAC pain scores; however, the reduction was significantly greater in the CurcuBoost® + SOC group than in the SOC-alone group (p = 0.0322). The corresponding reduction from baseline was also greater in the CurcuBoost® group (p = 0.0322). Additionally, the percentage reduction from baseline at week 4 was significantly greater in the CurcuBoost® group (38.9 ± 21.4%) than in the SOC group (25.7 ± 18.3%; p = 0.0126).

By week 8, the improvement in WOMAC pain was more pronounced. Participants receiving CurcuBoost® + SOC exhibited significantly lower pain scores compared with those receiving SOC alone (p < 0.0001). The reduction from baseline was significantly greater in the CurcuBoost® group compared with the SOC group, with a between-group mean difference of -3.04 (95% CI: -4.47 to -1.61), corresponding to a large effect size (Cohen’s d = 1.17). The percentage reduction from baseline to week 8 was also significantly greater in the CurcuBoost® group compared with the SOC group (p < 0.0001), indicating superior pain relief with CurcuBoost® supplementation over the eight-week treatment period.

Secondary efficacy outcomes

Secondary efficacy outcomes, including WOMAC stiffness, physical function, and total score, as well as EQ-5D, PGA, IGA, and VAS pain score, are summarized in Tables 3-6. Overall, participants receiving CurcuBoost® in addition to SOC demonstrated consistently greater improvements from baseline to week 8 across multiple efficacy domains compared with those receiving SOC alone, supporting a broad therapeutic benefit.

**Table 4:** VAS pain score (mITT population, n = 50) Data are presented as mean ± SD. p-values < 0.05 for ANCOVA were considered statistically significant. 95% CIs and Cohen's d values were calculated for between-group differences in change scores. "Δ" indicates change from baseline. SOC: standard of care; VAS: Visual Analog Scale, mITT: modified intention to treat, ANCOVA: analysis of covariance.

Time point/metric	CurcuBoost® + SOC	SOC alone	Mean difference (95% CI)	Cohen’s d	p-value
Baseline	7.72 ± 1.37	7.88 ± 1.36	-	-	-
Week 8	2.84 ± 0.80	4.76 ± 1.16	-	-	<0.0001
Δ week 8	-4.88 ± 1.72	-3.12 ± 1.54	-1.76 (-2.67 to -0.85)	1.08	<0.0001
% Δ week 8	-61.49 ± 14.88	-39.47 ± 18.30	-	-	<0.0001

**Table 5:** Patient and investigator assessments (mITT population, n = 50) Data are presented as mean ± SD. p-values < 0.05 for ANCOVA were considered statistically significant. 95% CIs and Cohen's d values were calculated for between-group differences in change scores. "Δ" indicates change from baseline. SOC: standard of care, mITT: modified intention to treat, ANCOVA: analysis of covariance.

Time point/metric	CurcuBoost® + SOC	SOC alone	Mean difference (95% CI)	Cohen’s d	p-value
Patient Global Assessment (PGA)					
Baseline	7.08 ± 1.22	6.80 ± 1.47	-	-	-
Week 8	2.88 ± 1.24	4.48 ± 1.98	-	-	0.0004
Δ week 8	-4.20 ± 1.44	-2.32 ± 2.06	-1.88 (-2.88 to -0.88)	1.06	0.0004
% Δ 8	-59.10 ± 16.63	-32.91 ± 29.46	-	-	0.0005
Investigator Global Assessment (IGA)					
Baseline	6.32 ± 1.60	6.88 ± 1.33	-	-	-
Week 8	2.56 ± 1.16	4.36 ± 1.55	-	-	0.0001
Δ week 8	-3.76 ± 1.54	-2.52 ± 1.53	-1.24 (-2.09 to -0.39)	0.81	0.0001
% Δ week 8	-59.01 ± 18.22	-35.86 ± 20.96	-	-	0.0001

**Table 6:** EQ-5D health-related quality of life (mITT population, n = 50) Data are presented as mean ± SD. p-values < 0.05 for ANCOVA were considered statistically significant. 95% CIs and Cohen's d values were calculated for between-group differences in change scores. "Δ" indicates change from baseline. SOC: standard of care, EQ-5D: EuroQol 5-Dimension, mITT: modified intention to treat, ANCOVA: analysis of covariance.

Time point/metric	CurcuBoost® + SOC	SOC alone	Mean difference (95% CI)	Cohen’s d	p-value
Baseline	49.40 ± 13.56	47.40 ± 11.91	-	-	-
Week 4	72.64 ± 6.65	57.32 ± 9.78	-	-	<0.0001
Week 8	81.72 ± 10.01	70.96 ± 13.01	-	-	0.0022
Δ week 4	+23.24 ± 16.87	+9.92 ± 13.80	-	-	<0.0001
Δ week 8	+32.32 ± 20.30	+23.56 ± 14.05	+8.76 (0.52-17.00)	0.50	0.0022
% Δ week 8	+83.27 ± 74.19	+57.51 ± 43.63	-	-	0.0009

WOMAC Stiffness

WOMAC stiffness improved in both groups (Table 3). By week 8, the mean reduction from baseline was numerically greater in the CurcuBoost® group (-3.84 ± 1.21) compared with SOC alone (-3.16 ± 1.40); however, the between-group difference did not reach statistical significance (mean difference: -0.68; 95% CI: -1.41 to 0.05; Cohen’s d = 0.52; p = 0.3161). Similarly, percentage reductions from baseline (-54.8% vs -47.4%) were not statistically different between groups (p = 0.1747).

WOMAC Physical Function

Participants receiving CurcuBoost® + SOC demonstrated significantly greater improvement in WOMAC physical function at week 8 than those receiving SOC alone (Table 3). The reduction from baseline was significantly greater in the CurcuBoost® group compared with the SOC group, with a between-group mean difference of -11.24 (95% CI: -16.84 to -5.64), corresponding to a large effect size (Cohen’s d = 1.12). Percentage improvement from baseline further supported this finding (-59.1% vs -38.9%; p < 0.0001).

WOMAC Total Score

Consistent with improvements observed across individual WOMAC domains, the WOMAC total score demonstrated significantly greater improvement in the CurcuBoost® + SOC group at week 8 (Table 3). By week 8, the reduction from baseline was significantly greater in the CurcuBoost® group compared with the SOC group, with a between-group mean difference of -14.96 (95% CI: -21.36 to -8.56), corresponding to a large effect size (Cohen’s d = 1.30; p < 0.0001). Percentage reductions were also significantly greater in the CurcuBoost® group (p < 0.0001).

VAS Pain Score

VAS pain scores improved significantly in both groups, with greater reductions in the CurcuBoost® + SOC group at week 8 (Table 4). By week 8, mean VAS scores decreased substantially in both groups (p < 0.0001). The reduction from baseline was significantly greater in the CurcuBoost® group compared with the SOC group, with a between-group mean difference of -1.76 (95% CI: -2.67 to -0.85), corresponding to a large effect size (Cohen’s d = 1.08). Percentage reductions were also significantly greater with CurcuBoost® (p < 0.0001), indicating superior pain relief.

Patient Global Assessment

Patient Global Assessment scores improved significantly more with CurcuBoost® + SOC than with SOC alone at week 8 (Table 5). The reduction from baseline was significantly greater with CurcuBoost® compared with SOC, with a between-group mean difference of -1.88 (95% CI: -2.88 to -0.88), corresponding to a large effect size (Cohen’s d = 1.06). Percentage reductions further supported this difference (p = 0.0005).

Investigator’s Global Assessment

Investigator-assessed global improvement also favored CurcuBoost® supplementation at week 8 (Table 5). Baseline IGA scores were similar between groups. By week 8, mean scores improved significantly in the CurcuBoost® group compared with SOC alone (p = 0.0001). The reduction from baseline was significantly greater in the CurcuBoost® group compared with the SOC group, with a between-group mean difference of -1.24 (95% CI: -2.09 to -0.39), corresponding to a large effect size (Cohen’s d = 0.81). Percentage reductions were also significantly greater in the CurcuBoost® group (p = 0.0001).

EQ-5D Health-Related Quality of Life

Health-related quality of life, assessed using the EQ-5D questionnaire, improved in both treatment groups, with significantly greater improvement observed in the CurcuBoost® group at week 8 (Table 6). Baseline EQ-5D scores were comparable between groups. By week 8, mean scores increased significantly in the CurcuBoost® group compared with SOC alone (p = 0.0022). The increase from baseline was greater in the CurcuBoost® group (+32.32 ± 20.30) compared with the SOC group (+23.56 ± 14.05), with a between-group mean difference of +8.76 (95% CI: 0.52 to 17.00), corresponding to a moderate effect size (Cohen’s d = 0.50). Percentage improvement was also significantly higher in the CurcuBoost® group (p = 0.0009).

OMERACT-OARSI responder analysis

As shown in Table 7, a greater proportion of participants receiving CurcuBoost® + SOC met the OMERACT-OARSI responder criteria than those receiving SOC alone. At week 4, responder rates were higher in the CurcuBoost® group (52% (n = 13) vs 28% (n = 7)), although the difference did not reach statistical significance (p = 0.0833). By week 8, responder rates increased to 80% (n = 20) in the CurcuBoost® group compared with 48% (n = 12) in the SOC group, representing a statistically significant between-group difference (p = 0.0184) and indicating a higher likelihood of achieving a clinically meaningful response with CurcuBoost® supplementation.

**Table 7:** OMERACT-OARSI responder analysis (mITT population, n = 50) Data presented as n (%). p-values < 0.05 for the chi-square test were considered statistically significant. SOC: standard of care, mITT: modified intention to treat.

Visits	Category	CurcuBoost® + SOC (N = 25)	SOC (N = 25)	χ² (df = 1)	Cramér’s V	p-value
Week 4	Responder	13 (52%)	7 (28%)	3.00	0.25	0.0833
	Non-responder	12 (48%)	18 (72%)			
Week 8	Responder	20 (80%)	12 (48%)	5.56	0.33	0.0184
	Non-responder	5 (20%)	13 (52%)			

Safety

**Figure 2:**
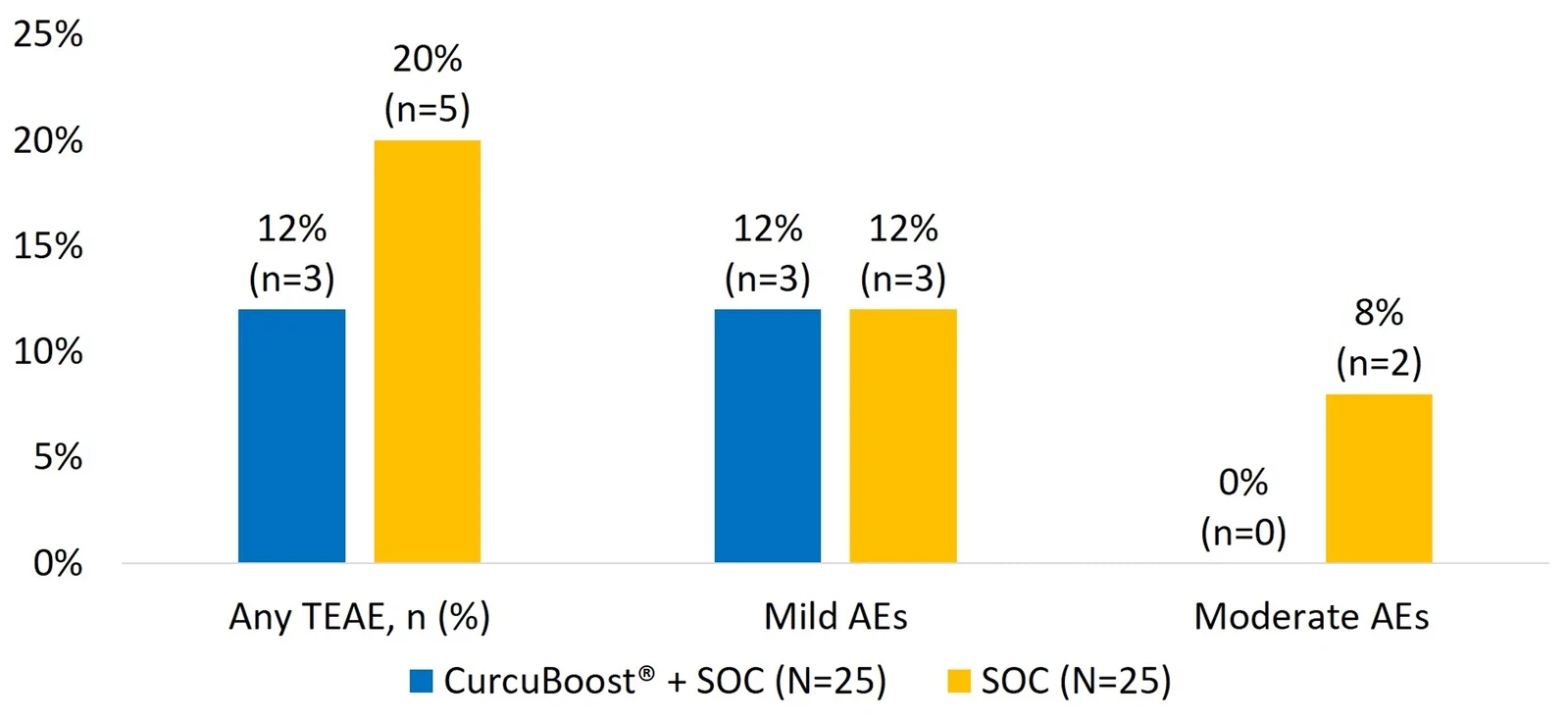
Treatment-emergent adverse events (safety population) Data are presented as % (n). AEs: adverse events, TEAEs: treatment-emergent adverse events, SOC: standard of care.

CurcuBoost® + SOC was well-tolerated (Figure 2). TEAEs occurred in three participants (12%) in the CurcuBoost® + SOC group and five participants (20%) in the SOC group. No drug-related TEAEs were reported, and no serious TEAEs or SAEs occurred in either group. All TEAEs were mild to moderate and resolved completely.

The most common TEAEs were headache, pain, pyrexia, and the common cold, all occurring at low frequencies and showing no meaningful between-group differences. Vital signs remained stable throughout the study, with no clinically significant abnormalities observed. Mean heart rate and temperature remained comparable between groups across all visits.

## Discussion

In this randomized, open-label clinical trial, adjunctive treatment with the bioavailability-enhanced curcumin formulation CurcuBoost® + SOC produced statistically and clinically meaningful improvements in subjects with knee OA compared with SOC alone. Reductions in WOMAC pain at week 8 were significantly greater with CurcuBoost® and were consistent across secondary outcomes, including WOMAC stiffness, physical function, and total scores; VAS pain; OMERACT-OARSI responder rates; global assessments; and EQ-5D. Improvements were evident by week 4 and sustained through week 8, with no increase in AEs, supporting favorable safety and tolerability.

Pain relief and functional improvement are central goals in knee OA management. The greater reductions in WOMAC and VAS pain scores observed with CurcuBoost® align with prior randomized trials and meta-analyses demonstrating curcumin's analgesic efficacy in knee OA [5,12,22,23]. Evidence indicates that curcuminoids significantly improve WOMAC and VAS scores compared with placebo or other controls and may be as effective as NSAIDs, with fewer adverse events [5,12,22,23]. Improvements in WOMAC stiffness, function, and total scores are also consistent with umbrella and systematic meta-analyses reporting benefits across multiple OA symptom domains [10,11]. These changes frequently exceed minimal clinically important differences, underscoring clinical relevance [11].

The higher proportion of OMERACT-OARSI responders further indicates meaningful composite improvement across pain, function, and global assessment domains. Improvements in EQ-5D and global assessments suggest broader benefits in quality of life, an increasingly important outcome in chronic OA management [6,24].

The observed effects are biologically plausible given curcumin’s anti-inflammatory and chondroprotective properties, including inhibition of NF-κB signaling, suppression of TNF-α and interleukin-1β, downregulation of cyclooxygenase-2, and reduction of matrix metalloproteinase activity [7,25,26]. Curcumin also mitigates oxidative stress and protects chondrocytes [27]. Meta-analyses report reductions in circulating inflammatory biomarkers, including C-reactive protein and TNF-α, with curcumin supplementation in knee OA [28]. Although biomarkers were not assessed here, symptomatic improvements are consistent with these mechanisms. Enhanced-bioavailability formulations achieve higher plasma concentrations than conventional extracts and have demonstrated improved clinical efficacy [29]. Although the pharmacokinetics of CurcuBoost® were not evaluated, the magnitude and consistency of the benefit suggest that improved systemic exposure may contribute to its effects.

CurcuBoost® was well tolerated, with no treatment-related serious AEs and a safety profile comparable to SOC alone. This is consistent with meta-analyses showing no significant increase in adverse events with curcuminoids and fewer gastrointestinal effects than NSAIDs [5,12,23]. Such tolerability is particularly relevant given the risks associated with long-term NSAID use in older adults and patients with comorbidities [5].

These findings support the use of bioavailability-enhanced curcumin as an effective adjunct in knee OA, providing meaningful symptom relief and quality-of-life improvements without compromising safety.

Limitations

Limitations include the open-label design, which may have introduced performance and detection bias because the primary and most secondary outcomes were patient-reported, and the eight-week study duration, which precluded assessment of long-term efficacy, structural outcomes, or sustained safety. In addition, although multiple secondary efficacy endpoints were evaluated, no formal multiplicity adjustment was prespecified. In addition, the standard-of-care regimen was individualized according to routine clinical practice rather than protocol-mandated, and detailed analyses of concomitant analgesic exposure or rescue medication use were not prespecified. Although identical SOC principles were applied in both treatment groups, the potential influence of differences in concomitant therapy cannot be completely excluded. The absence of pharmacokinetic and biomarker data limits mechanistic insight and may constrain generalizability. Future double-blind, longer-term, multicenter trials should confirm the durability of the benefit, assess inflammatory and pharmacokinetic parameters, and explore dose-response relationships to further define the role of enhanced curcumin formulations in knee OA.

## Conclusions

Adjunctive treatment with the bioavailability-enhanced curcumin formulation CurcuBoost® produced clinically meaningful improvements in pain, physical function, and quality of life in subjects with knee OA compared with SOC alone. Benefits across WOMAC, VAS, and responder outcomes were achieved without an increase in AEs, supporting a favorable safety profile. These findings reinforce the potential of curcumin with enhanced bioavailability as a complementary strategy in knee OA management and warrant confirmation in larger, longer-term studies.
